# A highly sensitive LC-MS/MS method for quantitative determination of 7 vitamin D metabolites in mouse brain tissue

**DOI:** 10.1007/s00216-023-04527-8

**Published:** 2023-01-27

**Authors:** Andrea Joy Stephenson, Benjamin Hunter, Paul Nicholas Shaw, Nur Sofiah Abu Kassim, Robert Trengove, Ryu Takechi, Virginie Lam, John Mamo

**Affiliations:** 1 Curtin Health Innovation Research Institute, Faculty of Health Sciences, Curtin University, Perth, WA Australia; 2 Curtin Medical School, Faculty of Health Sciences, Curtin University, Perth, WA Australia; 3 Separation Science & Metabolomics Laboratory at Murdoch University, Perth, Australia; 4 School of Pharmacy, The University of Queensland, Woolloongabba, QLD Australia; 5 School of Chemistry and Environment, Faculty of Applied Science, University Teknologi MARA, Negeri Sembilan, Cawangan Negeri Sembilan, Kampus Kuala Pilah, Kuala Pilah, Malaysia; 6 School of Population Health, Faculty of Health Sciences, Curtin University, Perth, WA Australia

**Keywords:** Brain, Vitamin D, 25(OH)D, 1,25(OH)D, 3-Epi-1,25(OH)D, 24,25(OH)D, Liquid chromatography tandem mass spectrometry

## Abstract

Despite its critical role in neurodevelopment and brain function, vitamin D (vit-D) homeostasis, metabolism, and kinetics within the central nervous system remain largely undetermined. Thus, it is of critical importance to establish an accurate, highly sensitive, and reproducible method to quantitate vit-D in brain tissue. Here, we present a novel liquid chromatography tandem mass spectrometry (LC-MS/MS) method and for the first time, demonstrate detection of seven major vit-D metabolites in brain tissues of C57BL/6J wild-type mice, namely 1,25(OH)_2_D_3_, 3-epi-1,25(OH)_2_D_3_, 1,25(OH)_2_D_2_, 25(OH)D_3_, 25(OH)D_2_, 24,25(OH)_2_D_3_, and 24,25(OH)_2_D_2_. Chromatographic separation was achieved on a pentaflurophenyl column with 3 mM ammonium formate water/methanol [A] and 3 mM ammonium formate methanol/isopropanol [B] mobile phase components. Detection was by positive ion electrospray tandem mass spectrometry with the EVOQ elite triple quadrupole mass spectrometer with an Advance ultra-high-performance liquid chromatograph and online extraction system. Calibration standards of each metabolite prepared in brain matrices were used to validate the detection range, precision, accuracy, and recovery. Isotopically labelled analogues, 1,25(OH)_2_D_3_-d_3_, 25(OH)D_3_-c_5_, and 24,25(OH)_2_D_3_-d_6_, served as the internal standards for the closest molecular-related metabolite in all measurements. Standards between 1 fg/mL and 10 ng/mL were injected with a resulting linear range between 0.001 and 1 ng, with an LLOD and LLOQ of 1 pg/mL and 12.5 pg/mL, respectively. The intra-/inter-day precision and accuracy for measuring brain vit-D metabolites ranged between 0.12–11.53% and 0.28–9.11%, respectively. Recovery in acetonitrile ranged between 99.09 and 106.92% for all metabolites. Collectively, the sensitivity and efficiency of our method supersedes previously reported protocols used to measure vit-D and to our knowledge, the first protocol to reveal the abundance of 25(OH)D_2_, 1,25(OH)D_2_, and 24,25(OH)_2_D_2_, in brain tissue of any species. This technique may be important in supporting the future advancement of pre-clinical research into the function of vit-D in neurophysiological and neuropsychiatric disorders, and neurodegeneration.

**Graphical Abstract:**

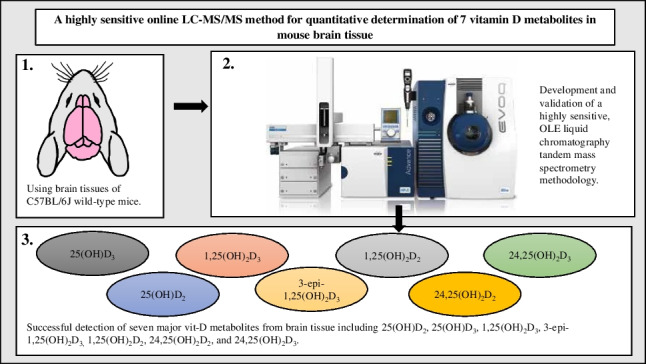

## Introduction

Increasing evidence indicates that vitamin D (vit-D) is a potent regulator of brain function. A number of epidemiological and experimental studies have implicated vit-D in a wide range of neurological functions including modulating neurotrophic factors, neuronal calcium regulation and signalling, neurotransmission, synaptogenesis, neurogenesis, and neuroprotection [1–5]. Moreover, disturbances in cerebral vit-D homeostasis have been linked to neurophysiological and neuropsychiatric disorders such as depression, autism, psychosis, and schizophrenia [6]. However, at present, the homeostasis, metabolism, and kinetics of vit-D metabolites in the brain remain largely unknown. Indeed, with techniques that are currently available, only three metabolites of vit-D—25(OH)D_3_, 24,25(OH)_2_D_3_, and 1,25(OH)_2_D_3_—have been reported in brain tissues to date [7–10]. Thus, it is important to establish an accurate and reproducible methodology to quantitate vit-D metabolites in brain.

The Vitamin D External Quality Assessment Scheme (DEQAS) compared methodologies for measuring 25(OH)D abundance in biological fluids such as plasma across 871 laboratories in 54 countries. DEQAS found that approximately 80% of laboratories used manual or automated immunoassays such as ELISA, whereas 20% used high-performance liquid chromatography (HPLC) or liquid chromatography with tandem mass spectrometry (LC-MS/MS) [11]. However, DEQAS and other studies reported high inter-assay and inter-laboratory variability amongst 25(OH)D metabolite immunoassays [12–18]. Immunoassays also require a significantly larger amount of sample in comparison to HPLC or LC-MS/MS, which often may not be suitable for small animal model studies, where sample volume is limited [19]. Furthermore, the possibility of cross-reactivity of the antibody due to the similarity in chemical structure is a source of potential incorrect results [20–22]. Immunoassays are generally not able to distinguish between 25(OH)D_2_ and 25(OH)D_3_ due to increased binding affinity to 25(OH)D_3_, reducing the assay detection sensitivities in comparison to HPLC, or LC-MS/MS [11]. The 24,25(OH)2D_3_ metabolite is present in approximately 10–15% of the total 25(OH)D concentration, thus significantly reducing the accuracy of immunoassays [23, 24] and possibly leading to aberrant interpretation of physiological effects.

The use of LC-MS/MS potentially enables for wide dynamic range quantitation of various compounds with small volumes, mitigating critical issues that may arise with immunodetection methods [25]. There have been many studies which have demonstrated the quantitative measurement of vit-D metabolites in blood (serum and plasma) using LC-MS/MS [26–34]; however, there is a paucity of studies which extract and analyse vit-D metabolites from lipid-rich brain tissue [7–10]. The recent review in 2022 by Alexandridou and Volmer of sample preparation techniques for vitamin D metabolites prior to LC-MS/MS analysis only cited one publication for brain tissue, human brain (now ref 31). Need of these limited brain focused LC-MS/MS studies, the methodologies identify limited metabolites and require further development. In 2014, Ahonen et al. developed a method utilising ultra-high-performance liquid chromatography-atmospheric pressure photoionisation-tandem mass spectrometry (UHLPC-APPI-MS/MS) to analyse vit-D-related compounds in mouse brain and cell line samples and were the first to report and quantify 25(OH)D_3_ in mouse brain [7]. In 2015, Xue et al. developed a method using high-performance liquid chromatography tandem mass spectrometry to simultaneously quantify the concentrations of 25(OH)D_3_ and 24,25(OH)_2_D_3_ in the brain of rats [8]. Fu et al. also developed an UHPLC-MS/MS method that successfully quantified 25(OH)D_3_ and 1,25(OH)_2_D_3_ in porcine brain and human brain [9]. However, the former protocol only reported the presence of 1,25(OH)_2_D_3_ in the prefrontal and middle frontal cortices and did not detect vit-D_3_ in any other regions of the brain. The method developed by Fu et al. [9] was used to study the −80 °C freezer storage stability of 25(OH)D_3_ in human brain [10]. The limitations across these protocols may be due to the use of complex and perhaps incomplete offline extractions, allowing the detection of only 1–2 metabolites at sensitivities in the ng/mL to pg/mL range. Moreover, C18 columns were used for each of the aforementioned methods, which may not be suitable for complex lipid-rich tissue separations such as brain.

In the present study, a highly sensitive method was developed, with a rapid automated online extraction (OLE) in conjunction with LC-MS/MS with a pentafluorophenyl (PFP) column. The protocol described enabled for the first time detection and quantification of seven major vit-D metabolites in mouse brain including 1,25(OH)_2_D_3_, 3-epi-1,25(OH)_2_D_3_, 1,25(OH)_2_D_2_, 25(OH)D_3_, 25(OH)D_2_, 24,25(OH)_2_D_3_, and 24,25(OH)_2_D_2_ with markedly greater sensitivity than reported protocols.

## Materials and methods

### Chemicals

All chemicals used in the protocol were LCMS grade. Acetonitrile (ACN), methanol, and water were purchased from Thermo Fisher (MA, USA). Ammonium formate (> 99%) was purchased from Sigma-Aldrich (Darmstadt, Germany). All standard vit-D compounds and isotope-labelled analogues, 1,25(OH)_2_D_3_, 1,25(OH)_2_D_2_, 24,25(OH)_2_D_3_, 24,25(OH)_2_D_3_-d_6_, 24,25(OH)_2_D_2_, 1,25(OH)_2_D_3_-d_3_, 25(OH)D_3_, 25(OH)D_2_, and 25(OH)D_3_-c_5_, were purchased from PM Separations Pty Ltd (QLD, Australia) and from IsoSciences, Ambler, PA (USA). The 3-Epi-1,25(OH)_2_D_3_ was purchased from abcr, Germany.

### Mouse brain sample collection and preparation

Twelve male C57BL/6J mice aged 6 weeks were obtained from the Animal Resource Centre (Murdoch, WA). The animals were maintained in an environmentally controlled animal facility in ventilated cages, temperature 22 °C, 12-h light/dark cycle (Curtin Animal Holding Facility, Curtin University). They were fed standard laboratory chow (AIN93M, Speciality Feeds, Glen Forrest, WA) with water ad libitum. Animals were upheld in accordance with internationally accepted ethical principles for laboratory animal use. All procedures described in this study abided by the Animal Research: Reporting of In Vivo Experiments (ARRIVE) guidelines and were approved by Curtin Animal Ethics Committee (approval no. ARE2018-35).

At 7 weeks of age, the mice were anaesthetised with 2% gaseous isoflurane and euthanised via cervical dislocation [35]. Whole-brain specimens were carefully extracted and immediately snap frozen in liquid nitrogen and kept at −80 °C until analysis. Whole-brain samples were thawed and weighed before being manually ground with ACN using a small pestle. The optimised ACN volume used for metabolite extraction was brain weight (g) divided by 0.15. Three isotopically labelled standards (25(OH)D_3_-^13^C_5_, 1,25(OH)_2_D_3_-d_3_, 24,25(OH)_2_D_3_-d_6_) were added for a final concentration of 1.25 ng/mL. The brain homogenate was sonicated in a water bath at 4 °C (30-s cycles of 10 s on/20 s off) for 15 min and incubated at 4 °C for 10 min to facilitate protein precipitation. The sample was then vigorously vortexed for 5 min at 22 °C and centrifuged at 1500 relative centrifugal force (×*g*) for 10 min at 4 °C. The supernatant from 12 individual mice was then pooled. The sample was stored at −80 °C until use. Two hundred microliters of 3 mM ammonium formate in H_2_O was added followed by 1-min vortex immediately prior to analysis.

All following validation analyses were done by spiking vit-D metabolite standards into brain homogenate in order to validate the practicality and applicability of our methods in the mouse brain tissue matrix. The validation analyses were done by subtracting the concentrations of endogenous vit-D metabolites in the blank brain matrix.

### Online extraction

**Fig. 1:**
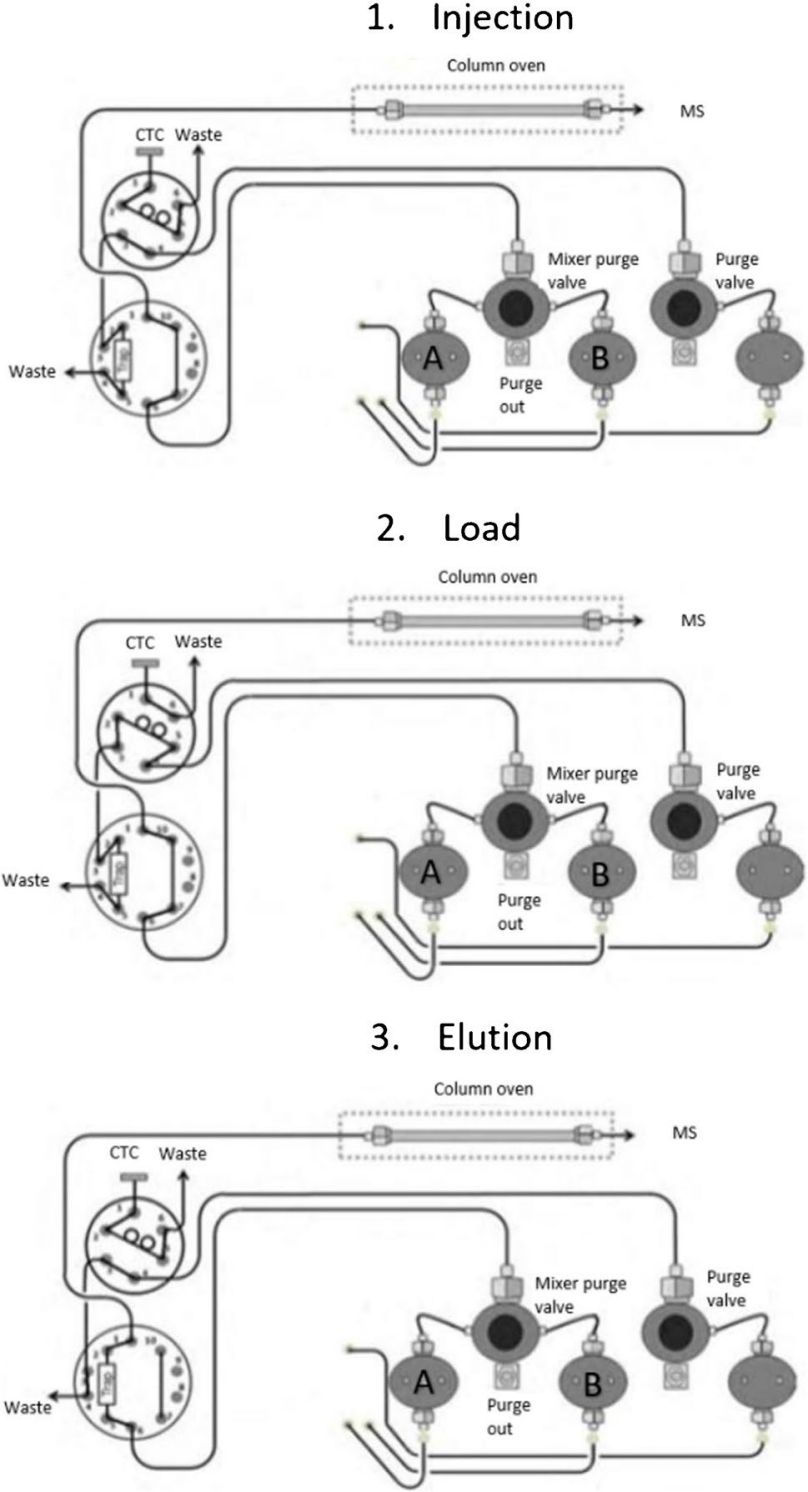
OLE configuration

All standards and samples were run with an online extraction method in order to increase efficiency and throughput whilst simplifying the workflow. Online extraction was performed using a 6-port injection valve with a 500-μL loop and a 10-port switching valve. Flow connections and valve programming are presented in Fig. 1. The sample is injected into the loop in valve position 1 and loaded onto the trap column in valve position 2. This cycle can be repeated as many times as necessary to concentrate the sample before, with valves in position 3, and the analyte is eluted from the trap column onto the analytical column. A Poroshell 120 EC-C18 guard column (2.1 × 5 mm, 2.7 µm; Agilent Technologies, Santa Clara, CA, USA) was used to trap vit-D metabolites, before back flushing onto the analytical column. The trap column was equilibrated at 1 mL/min for 1 min with 50% methanol with 3 mM ammonium formate in water:methanol (50:50) using pump C, then 80 μL of prepared brain sample was injected into the loop (valves in position 1) and then loaded onto the trap (valves in position 2) for 45 s at 1 mL/min. This cycle (valve position 1, then valve position 2) was repeated for a total of 5 trapping cycles, resulting in a total sample volume injection of 400 μL. Re-equilibration of the trap column occurred simultaneously with the re-equilibration of the analytical column using pump C, and therefore did not affect the analysis time.

### Determination of retention time and collision energies of vit-D precursor and product pairs

Retention time and collision energies were determined via direct injection and infusion of the vit-D standards and isotopically labelled standards. Each vit-D metabolite infusion solution, 1,25(OH)_2_D_3_, 1,25(OH)_2_D_2_, 24,25(OH)_2_D_3_, 24,25(OH)_2_D_3_-d_6_, 24,25(OH)_2_D_2_, 1,25(OH)_2_D_3_-d_3_, 25(OH)D_3_, 25(OH)D_2_, and 25(OH)D_3_-^13^C_5_, was prepared by dissolving in ethanol at 1 mg/mL concentration, pipetted into aliquots, and stored in amber glass vials at −80 °C. All working solutions were prepared by serial dilution of the stock solutions in ACN. For the determination of retention time and collision energies, 100 ng/mL standard concentrations were used.

### LC-MS/MS analysis

Vit-D metabolites were analysed using the EVOQ elite triple quadrupole mass spectrometer with an Advance UHPLC OLE system (Bruker, Billerica, MA) and a CTC HTS-xt autosampler (CTC Analytics AG, Switzerland), where the samples resided in glass vials at 4 °C. The mass spectrometer was operated in MS/MS mode with HESI source, capillary voltage set to 3500 V, cone at 350 °C and 20 psi gas, heated probe at 300 °C and 50 psi, and nebuliser at 60 psi (Table 1). Analyses were performed using a Poroshell 120 pentafluorophenyl (PFP) column (150 × 2.1 mm i.d., 2.7-µm particle size; Agilent Technologies, Santa Clara, CA, USA) with the column oven set to 50 °C. Brain homogenate injection volumes were 80 µL. Mobile phase A was 3 mM ammonium formate in water:methanol (50:50) and mobile phase B was 3 mM ammonium formate in methanol:isopropanol (50:50).(Tables 2 and 3).

**Table 1:** Liquid chromatography programming

Time (min)	Pump B (%)
0	50
3	50
15	90
15.1	100
35.1	100
35.2	50
40.2	50

**Table 2:** Precursor and product pairs and associated retention time and collision energies

Vitamin D metabolite and adduct	Precursor	Products	Retention time (min)	Collison energy (V)
1,25(OH)_2_D_3_ [M+H-H_2_O]^+^	399.3	381.2	7.02	9.0
		135.1		21.0
1,25(OH)_2_D_2_ [M+NH_4_]^+^	446.3	411.3	7.36	9.0
		135.1		11.0
1,25(OH)_2_D_3_-d_3_ [M+NH_4_]^+^	437.3	384.3	7.03	8.0
		138.1		17.0
24,25(OH)_2_D_3_ [M+H]^+^	417.3	381.3	6.48	8.0
		399.3		7.0
24,25(OH)_2_D_2_ [M+H-2H_2_O]^+^	393.3	268.1	7.02	24.0
		243.2		9.0
24,25(OH)_2_D_3_-d_6_ [M+H]^+^	423.3	405.2	6.47	9.0
		387.2		13.0
25(OH)D_3_ [M+H]^+^	401.30	383.3	8.98	7.0
		365.3		8.0
25(OH)D_2_ [M+H]^+^	413.3	395.3	9.20	8.0
		107.2		21.0
25(OH)D_3_-c_5_ [M+H]^+^	406.2	388.2	8.97	8.0
		370.2		7.0

**Table 3:** Lower limit of detection (LLOD), lower limits of quantification (LLOQ), and linear range of exogenous standards, using a maximum of ten measuring points between 1 fg/mL and 10 ng/mL

Vit-D metabolite	LLOD (pg/mL)	LLOQ (pg/mL)	Linear range (pg/mL)
1,25(OH)_2_D_3_	1	12.5	1–1000
1,25(OH)_2_D_2_	1	12.5	10–1000
24,25(OH)_2_D_3_	1	12.5	10–1000
24,25(OH)_2_D_2_	1	12.5	10–1000
25(OH)D_3_	1	12.5	10–1000
25(OH)D_2_	1	12.5	10–1000

### Linearity and lower limits of detection/quantification

The calibration curve for each vit-D metabolite was drawn and the LLOD/LLOQ was determined using vit-D standards ranging from 1 fg/mL to 10 ng/mL. The calibration standards were prepared by diluting the stock solution in ACN and adding this to the brain matrix and then analysed by LC-MS/MS coupled with online extraction. All of the standards could be detected over the complete range 1 fg/mL to 10 ng/ml, whilst for concentrations below 1 pg/mL, the response was systematic but generally with a curved shape rather than linear, and this is reflected in the non-linearity of S/N in the low concentration range. As a consequence, conservative and universal LLOD, LLOQ, and linearity range values are given in Table 4, based on examination of linearity over all ranges of concentration.

**Table 4:** Average signal to noise ratio (S/N) for the six vitamin D metabolites, over the linear concentration range, measured over 3 days for exogenous brain homogenate

1 pg/mL	5121.3	853.0	444.3	272.7	62.0	336.0
10 pg/mL	5929.0	7301.3	639.7	393.3	702.0	1234.0
100 pg/mL	12,495.0	30,369.7	939.3	2188.3	3222.0	8168.0
500 pg/mL	15,806.3	31,598.3	1778.3	7225.0	5167.7	17,775.3
1000 pg/mL	18,377.0	61,069.0	2267.0	14,995.0	6305.0	46,980.0

### Intra- and inter-day precision and accuracy

The reproducibility of intra- and inter-day measurements was determined with the brain matrix spiked with low, medium, and high standard solutions at concentrations of 100, 500, and 1000 pg/mL, respectively. Intra-day precision and accuracy were determined with 3 replicate measurements of each low, medium, and high standards. Inter-day precision and accuracy were determined with repeated measures of low, medium, and high standards over 3 consecutive days. Metabolites were quantified by normalisation to the closest matching internal standard and subsequent calculation from the constructed matching calibration curves. The same application was used in the quantification of 1,25(OH)_2_D_3_, 1,25(OH)_2_D_2_, 25(OH)D_3_, 25(OH)D_2_, 24,25(OH)_2_D_3_, and 24,25(OH)_2_D_2_.

Precision was calculated as the total variance of quantified results, whilst accuracy was determined as the difference between the actual and calculated theoretical concentrations. Precision and accuracy were calculated in both intra- and inter-day periods and expressed as a percentage relative standard deviation (RSD%).

### Recovery

The recovery rate of vit-D metabolites during the extraction process was determined by spiking 400 μL of ACN with 10 μL of vit-D standard solution (100 ng/mL) before or after the extraction using the mouse brain procedure described in the “Mouse brain sample collection and preparation”, then comparing the difference in concentrations for the pre-extraction spike and the post-extraction spike. The measurements were repeated in triplicate and are reported as a percentage of the pre-extraction spike results.

### Application of the established method to the measurement of endogenous cerebral vit-D metabolites

The concentrations of endogenous brain vit-D metabolites, 1,25(OH)_2_D_3_, 1,25(OH)_2_D_2_, 25(OH)D_3_, 25(OH)D_2_, 24,25(OH)_2_D_3_, and 24,25(OH)_2_D_2_, were measured in brain tissue homogenate samples spiked with isotopically labelled internal standards. The brain matrix was run on LC-MS/MS with online extraction as described above. The concentration of each metabolite was concurrently determined based on a calibration curve. Seven replicates were run daily, over 3 days.

### Statistical analysis

Linearity, slope, and regression coefficients were determined by linear regression. Microsoft Excel 2010 was used for all statistical analyses.

## Results

### Determination of retention time and collision energy

**Fig. 2:**
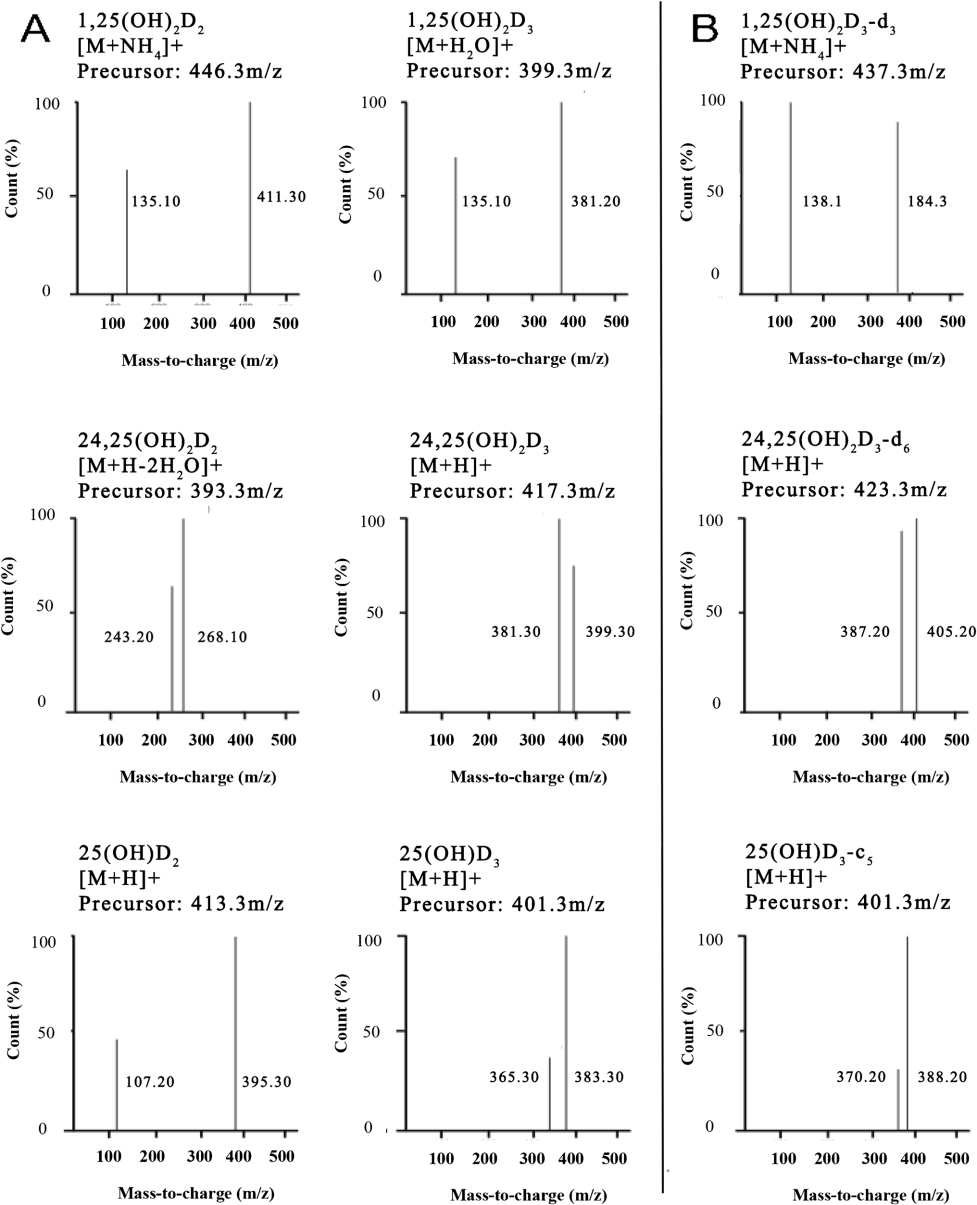
Precursor/product ion spectrum of six vit-D metabolites (**A**) and associated isotopically labelled internal standards (**B**) in mouse brain

**Fig. 3:**
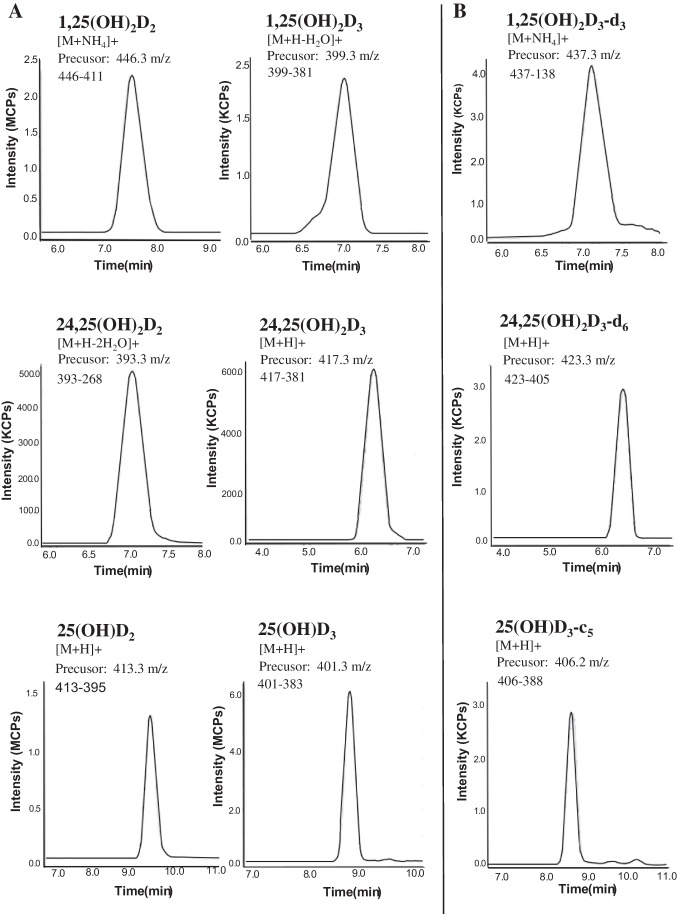
Chromatograms of the quantitative transition signal for the six vit-D metabolite standards (**A**) and the chromatograms of the associated isotopically labelled internal standards (**B**) in mouse brain at a concentration of 100 ng/mL, with a mobile phase flow rate of 300 µL/min

The retention time and collision energy of each vit-D metabolite were determined by using vit-D standards. Targeted analytes produced a much stronger signal in positive ion mode using the electrospray ion source; thus, all the vit-D metabolites were detected under MRM positive mode. The most abundant precursor ions were chosen to increase sensitivity whilst product ions were selected according to greatest intensity and optimal sensitivity. Precursor and product pairs, associated retention times and collision energy, are presented in Table 2 and shown in Fig. 2. The OLE-LC-MS/MS method shows good chromatographic performance with all eluting analytes showing clear separation with confident individual detection (Fig. 3).

### Linearity, limits of detection, and quantification

Calibration standards ranging from 1 fg/mL to 10 ng/mL were used to determine the method linear range, LLOD, and LLOQ and these are listed in Table 3. The average signal to noise (S/N) for each metabolite at each concentration over the linear range is shown in Table 4. Vitamin D standards spiked into brain matrix were detectable as low as 500 fg/mL and quantifiable as low as 12.5 pg/mL. The linear range was only assessed to a maximum of 10 ng/mL for all metabolites. All metabolites displayed a curved response as a function of concentration at the lower concentrations as the result of matrix effects and this is reflected in apparent lack of linearity at the lower concentrations and the resulting S/N table.

### Intra- and inter-day repeatability and recovery

Intra-day precision and accuracy (RSD%) for vit-D metabolite standards in brain matrix are presented in Table 5. The accuracy ranged from between 80 and 120% of the spiked concentrations, and the precision was less than 20% except for 25(OH)D_2_ at 1000 ng/mL. The precision in the inter-day measures for all metabolites was between 12.47 and −9.53% (Table 6). The recovery ranged between 99.06 and 106.92% (Table 7).

**Table 5:** Intra-day accuracy and precision (RSD%)

Concentration, pg/mL	Vitamin D metabolite	Day 1		Day 2		Day 3	
		Accuracy, RSD%	Precision, RSD%	Accuracy, RSD%	Precision, RSD%	Accuracy, RSD%	Precision, RSD%
100	1,25(OH)_2_D_3_	2.35	3.97	−16.96	4.23	5.89	6.04
	1,25(OH)_2_D_2_	−9.37	2.29	8.18	7.25	−9.58	0.82
	24,25(OH)_2_D_3_	3.56	4.40	4.15	7.23	10.57	6.65
	24,25(OH)_2_D_2_	−6.76	5.11	−6.76	4.77	12.93	11.33
	25(OH)D_3_	3.51	3.49	4.29	13.89	11.04	2.82
	25(OH)D_2_	14.23	11.73	4.76	22.32	17.66	3.35
500	1,25(OH)_2_D_3_	−12.06	9.59	−9.70	1.00	−6.83	4.33
	1,25(OH)_2_D_2_	−8.81	2.79	7.25	6.4	−12.12	2.73
	24,25(OH)_2_D_3_	−17.35	1.88	9.40	4.20	−4.53	1.03
	24,25(OH)_2_D_2_	−16.68	1.93	−16.68	2.96	6.81	1.77
	25(OH)D_3_	−6.80	5.51	14.69	7.73	−3.87	2.05
	25(OH)D_2_	−3.52	4.52	13.84	5.45	−2.51	3.21
1000	1,25(OH)_2_D_3_	12.87	11.64	−3.84	0.98	5.61	16.10
	1,25(OH)_2_D_2_	8.67	7.08	−15.97	6.04	−15.01	19.50
	24,25(OH)_2_D_3_	−1.39	5.44	17.66	22.05	−31.57	4.13
	24,25(OH)_2_D_2_	−0.41	5.37	−0.41	4.38	10.44	6.80
	25(OH)D_3_	2.36	7.00	14.91	4.03	−18.02	16.33
	25(OH)D_2_	12.90	7.58	18.23	5.16	−2.11	22.06

**Table 6:** Inter-day accuracy and precision (RSD%)

Concentration, pg/mL	Vitamin D metabolite	Inter-day	
		Accuracy, RSD%	Precision, RSD%
100	1,25(OH)_2_D_3_	−2.90	4.75
	1,25(OH)_2_D_2_	−3.59	3.45
	24,25(OH)_2_D_3_	6.10	6.09
	24,25(OH)_2_D_2_	−0.20	7.07
	25(OH)D_3_	6.28	6.73
	25(OH)D_2_	12.22	12.47
500	1,25(OH)_2_D_3_	−9.53	4.97
	1,25(OH)_2_D_2_	−4.56	3.98
	24,25(OH)_2_D_3_	−4.16	2.37
	24,25(OH)_2_D_2_	−8.85	2.22
	25(OH)D_3_	1.34	5.10
	25(OH)D_2_	2.60	4.47
1000	1,25(OH)_2_D_3_	4.88	9.58
	1,25(OH)_2_D_2_	−7.44	10.88
	24,25(OH)_2_D_3_	−5.10	10.54
	24,25(OH)_2_D_2_	−3.0	5.52
	25(OH)D_3_	−0.25	9.12
	25(OH)D_2_	4.47	11.60

**Table 7:** Recovery difference (%) of metabolites for the prespike and postspike extraction procedure in acetonitrile matrix showing mean, SD, and RSD (%)

		1,25(OH)_2_D_2_	1,25(OH)_2_D_3_	24,25(OH)_2_D_3_	24,25(OH)_2_D_2_	25(OH)D_2_	25(OH)D_3_
Prespike	Mean	5.32	0.15	2.17	1.73	1.32	11.89
	SD	0.31	0.00	0.08	0.05	0.04	0.50
	RSD%	5.75	2.20	3.53	3.15	3.34	4.18
Postspike	Mean	5.76	0.15	2.32	1.71	1.36	12.71
	SD	1.54	0.04	0.28	0.32	0.43	1.33
	RSD%	26.73	28.44	11.99	18.78	31.69	10.47
	Recovery % (postspike/prespike)*100	108.32	105.39	106.60	99.09	103.01	106.92

### Measurement of endogenous brain vit-D metabolites

Mean metabolite concentrations, standard deviation, and intra- and inter-day precision (RSD%) measurements for endogenous vit-D are presented in Table 8. Intra- and inter-day precision for all metabolites ranged between 0.12–11.53% and 0.28–9.11%, respectively.

**Table 8:** Mean, standard deviation, and RSD% measures of endogenous homogenate (pg/mL)

		1,25(OH)_2_D_2_	1,25(OH)_2_D_3_	24,25(OH)_2_D_2_	24,25(OH)_2_D_3_	25(OH)D_2_	25(OH)D_3_
Intra-day							
Day 1	Mean, pg/mL	24.99	ND	234.77	91.54	127.93	19.72
	SD	0.42		15.32	0.11	0.80	0.34
	% RSD	1.68		6.53	0.12	0.62	1.70
Day 2	Mean, pg/mL	25.19	ND	205.48	92.05	114.85	18.43
	SD	0.42		23.69	0.22	0.87	0.31
	% RSD	1.67		11.53	0.23	0.76	1.66
Day 3	Mean, pg/mL	24.49	ND	222.66	92.78	143.41	13.77
	SD	0.23		20.65	0.44	4.43	1.41
	% RSD	0.94		9.27	0.48	3.09	10.21
Inter-day							
Average	Mean, pg/mL	24.89	ND	220.97	92.12	128.73	17.31
	SD	0.36		19.89	0.26	2.03	0.68
	% RSD	1.43		9.11	0.28	1.49	4.53

Five vit-D metabolites were detected endogenously within the validated detection range. These include 1,25(OH)_2_D_2_, 25(OH)D_3_, 25(OH)D_2_, 24,25(OH)_2_D_3_, and 24,25(OH)_2_D_2_, whilst 1,25(OH)_2_D_3_ was not detected in the brain (Table 8) at the validated retention time.

#### Detection and validation of 3-epi-1,25(OH)_2_D_3_ metabolite

**Fig. 4:**
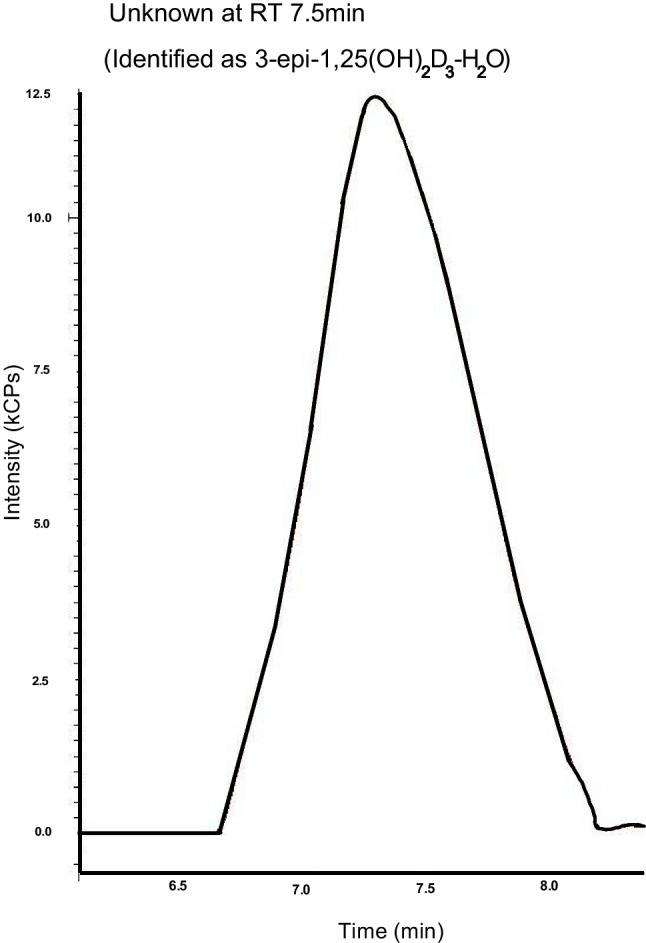
An unknown 7.5-min peak was observed with the 1,25(OH)_2_D_3_ transitions (399.3 > 135.2) for retention time 7.02 min later validated as 3-epi-1,25(OH)_2_D_3_

All analytes were scanned for over the full retention time of the method during method development and an unknown peak was observed in the chromatogram at 7.5 min as shown in Fig. 4 for the 1,25(OH)_2_D_3_ transitions 399.3 > 135.1 and 399.3 > 381.2 and these transitions were used to semi-quantitate the 7.5-min retention time compound (Table 9 and Table 10). After a literature search, this was proposed to be the epimer of 1,25(OH)_2_D_3_ (3-epi-1,25(OH)_2_D_3_). A standard for the epimer (abcr, Germany cas 61476-45-7) was ordered and used to confirm the 7.5-min retention time and the MRM transitions determined for the epimer (Table 11). The 3-epi-1,25(OH)_2_D_3_ exhibited a precursor of 399.3 m/z, corresponding to the loss of H_2_O and a precursor of 434.3 corresponding to an ammonium adduct, and both formed a m/z 135.1 product ion and a m/z 381.2 product ion, although at lower intensity

**Table 9:** 3-Epi-1,25(OH)_2_D_3_ retention time validation

3-Epi-1,25(OH)_2_D_3_				
Solution	Sample name	Retention time (min)	Peak area	Signal/noise ratio
Spiked acetonitrile (1.25 ng)	Standard 1	7.542	4356	61
	Standard 2	7.615	2084	100
	Standard 3	7.495	3510	55
	Standard deviation	**0.06**		
	Mean	**7.55**		
	% RSD	**0.80**		
Spiked brain homogenate (1.25 ng)	Endogenous 1	7.52	1797	15
	Endogenous 2	7.64	1820	18
	Endogenous 3	7.66	3548	31
	Standard deviation	**0.07**		
	Mean	**7.61**		
	% RSD	**0.95**		
Spiked brain homogenate calibration (ng)	1.6	7.47	7094	59
	3.125	7.39	21,260	140
	6.25	7.43	54,577	260
	12.5	7.42	120,320	860
	25	7.40	267,640	1432
	50	7.40	526,309	2164
	100	7.31	1,003,098	2206
	Standard deviation	**0.04**		
	Mean	**7.36**		
	% RSD	**0.58**		
Average retention time, standard deviation		**0.05**		
Average retention time, mean		**7.50**		
Average retention time, % RSD		**0.77**		

This table provides parameters of an unknown peak we later validated and identified as the 3-epi-1,25(OH)2D3 metabolite

**Table 10:** Mean, standard deviation, and RSD% measures of endogenous 3-epi-1,25(OH)_2_D_3_ homogenate (pg/mL)

3-Epi-1,25(OH)_2_D_3_			
	Mean (pg/mL)	SD	RSD%
Intra-day			
Day 1	227.09	19.38	8.53
Day 2	228.21	18.92	8.29
Day 3	268.27	6.79	2.53
Inter-day			
Average	241.19	15.03	6.45

**Table 11:** 3-Epi-1,25(OH)_2_D_3_ precursor and products, retention time, and collision energy

Precursor	Products	Retention time (min)	Collison energy (V)
399.20	199.10	7.50	13.00
	299.10		9.00

We identified an unknown, but consistent peak at 7.5 min in the 1,25(OH)_2_D_3_ chromatogram (Fig. 4). We validated this as the epimer of 1,25(OH)_2_D_3_ as per the protocol described in the “Mouse brain sample collection and preparation” using a 3-epi-1,25(OH)_2_D_3_ standard (abcr, Germany cas 61476-45-7).

3-Epi-1,25(OH)2D3 optimised precursor and product pairs and collision energy are listed in Table 9. To validate the retention time, spiked known concentrations (1.25 ng**/**mL) of 3-epi-1,25(OH)_2_D_3_ standard were run in both ACN and brain matrix, and brain matrix calibration (1.6–100 ng**/**mL) confirming the 7.5-min retention time (Table 10).

Mean concentrations, standard deviation, and intra- and inter-day precision (RSD%) measures for validated endogenous 3-epi-1,25(OH)_2_D_3_ are presented in Table 11. Intra-day precision for 3-epi-1,25(OH)_2_D_3_ ranged between 2.53 and 8.53%, and inter-day precision was 6.45%.

## Discussion

This study describes a rapid, sensitive OLE-LC-MS/MS protocol on PFP columns that separates and quantifies the seven vit-D metabolites, 1,25(OH)_2_D_3_; 3-epi-1,25(OH)_2_D_3_; 1,25(OH)_2_D_2_; 25(OH)D_3_; 25(OH)D_2_; 24,25(OH)_2_D_3_; and 24,25(OH)_2_D_2_, in mouse brain.

Previous studies reporting vit-D metabolites in the brain have used offline extraction techniques requiring extensive overnight derivatisation, solid-phase extraction, and liquid-liquid extraction, to quantify vit-D metabolites with LCMS methods [7–10]. We extend those studies and describe herein a simplified, more sensitive, and robust online extraction process, enabling a stacked loading protocol to effectively concentrate the abundance of quantification of vit-D metabolites in the brain. The protocol detailed addresses potential significant confounders and achieves markedly improved sensitivity and identification of brain vit-D metabolites that may be physiologically important.

C18 columns have been historically used in LCMS protocols to determine vit-D metabolites [7–9]. The C18 matrix has preferential retention of hydrophobic moieties within the stationary phase, notionally enabling detection of vit-D metabolites[36]. However, commonly residual abundance of brain phospholipids from offline extracts with direct loading thereafter to C18 columns may markedly interfere with target vit-D metabolite detection and/or significantly increase the threshold detection requirements [37].

The online extraction component of the novel OLE-LC-MS/MS method described with selective elution from the trap column enables effective bulk decontamination of phospholipids [38]. With the option to loop load and stack, effectively concentrating metabolites of interest, coupled with phospholipid removal, sensitivity appears to be improved by orders of magnitude and other metabolites of vit-D with potentially important biological function are realised. Indeed, we report new measures of less-polar metabolites of vit-D in mouse brains using the OLE-LCMS on PFP. We suggest that the rigidity of the PFP endcapping provides better shape selectivity, cation exchange, charge transfer, electrostatic interactions, hydrogen bonding, and dipole and pi-pi interactions, which provides greater separation of structural isomers of vit-D compared to C18 reversed phase methods [39].

In the protocol detailed, solvents were selected to utilise the greater hydrogen bonding capabilities of the PFP column compared to C18 columns. We report that water:methanol:isopropanol (2:49:49) with 3 mM ammonium formate obtained the highest recovery for all 7 vit-D metabolites reported. Different concentrations of ammonium formate (3 mM, 6 mM, and 10 mM) were tested during the method development processes and 3 mM was chosen for the maximum yield. The method described had excellent limits of detection and quantification with 500 fg/mL and 1 pg/mL respectively. Furthermore, recovery rate of all metabolites ranged between 99.09 and 108.32%. Collectively, this supports the selectivity and sensitivity of the protocol described.

The method detailed here demonstrates a high rate of accuracy and is congruent with previous methods being well within the general range of 80–120% [7]. Considering our wide detection range in complex brain matrix, the results indicate appropriate accuracy of the method by means of repeatability. Furthermore, our targeted vit-D cerebral metabolic profiling method removes any unwarranted sample extraction procedure providing unbiased and robust results, with minimum sample preparation.

## Conclusions

The critical role of vit-D in central nervous system function and evidence of an association with some neurological disorders and neurodegenerative diseases is increasingly indicated. Indeed, the active form of vit-D, 1,25(OH)D_3_, and key enzymes for vit-D metabolism such as CYP27B1 have been identified in cells of the central nervous system. However, only three vit-D metabolites have been reported in brain tissue specimens (25(OH)D_3_, 24,25(OH)_2_D_3_, and 1,25(OH)D_3_) previously. Clearly, more robust and sensitive methods than are currently available are required to understand cerebral vit-D homeostasis and if this is associated with brain function.

This study validates a relatively simple, sensitive, rapid, and specific OLE-LC-MS/MS method to screen and quantify vit-D brain metabolites in the brain of C57BL/6J mice. This present study has expanded the vit-D metabolites than can be analysed in brain tissue, at lower concentrations, and provides the basis for future studies comparing vit-D metabolism in brain of different mice strains developed to study neurological disorders and diseases.

## Contributions to the field statement

Current and emerging data collected from experimental, pre-clinical, and clinical studies have demonstrated disturbances in serum vit-D homeostasis as a major risk in the development and progression neurodegenerative disorders. Whilst the detrimental impact of vit-D deficiency throughout the peripheral and central nervous system is well established, little is known regarding the impact of vit-D dyshomeostasis within the brain. At present, the reference range for vit-D serum levels are set to maintain skeletal health, whilst an appropriate concentration for vit-D within the central nervous system remain to be elucidated. Classification of what vit-D levels are recommended is ambiguous and controversial, whilst any mechanisms for potential adverse effects of exaggerated vit-D levels on CNS function remain to be investigated. This protocol significantly improves currently available method of cerebral vit-D detection, developed to the use of the OLE-LC-MS/MS method with PFP column. This expanded the detectable range of vit-D metabolites to fg/mL range, which is ~1000-fold improvement. It demonstrates cerebral detection of 7 vit-D metabolites, as opposed to the two previously reported. This is critical in allowing researchers to explore both the fundamental role of vit-D synthesis, metabolism, and homeostasis within the brain, allowing us to further our understanding into neurological and neurodegenerative human pathologies.

## References

[1] Groves NJ, McGrath JJ, Burne TH (2014). Vitamin D as a neurosteroid affecting the developing and adult brain. Annu Rev Nutr.

[2] Garcion E (2002). New clues about vitamin D functions in the nervous system. Trends Endocrinol Metab.

[3] Cui X (2015). Vitamin D and the brain: key questions for future research. J Steroid Biochem Mol Biol.

[4] Kalueff AV, Eremin KO, Tuohimaa P (2004). Mechanisms of neuroprotective action of vitamin D(3). Biochemistry (Mosc).

[5] Lam V (2015). An investigation of a vitamin D, ionised calcium and parathyroid hormone regulatory axis of cerebral capillary function: implications for cognitive performance in ageing. Plos One.

[6] Anjum I (2018). The role of vitamin D in brain health: a mini literature review. Cureus.

[7] Ahonen L (2014). Analysis of oxysterols and vitamin D metabolites in mouse brain and cell line samples by ultra-high-performance liquid chromatography-atmospheric pressure photoionization–mass spectrometry. J Chromatogr A.

[8] Xue Y (2015). Simultaneous quantification of 25-hydroxyvitamin D3 and 24,25-dihydroxyvitamin D3 in rats shows strong correlations between serum and brain tissue levels. Int J Endocrinol.

[9] Fu X (2019). Determination of vitamin D and its metabolites in human brain using an ultra-pressure LC-tandem mass spectra method. Curr Dev Nutr.

[10] Fu X (2021). Vitamin D and vitamin K concentrations in human brain tissue are influenced by freezer storage time: the memory and aging project. J Nutr.

[11] Dirks NF (2018). The when, what & how of measuring vitamin D metabolism in clinical medicine. Nutrients.

[12] Carter G (2004). How accurate are assays for 25-hydroxyvitamin D? Data from the International Vitamin D External Quality Assessment Scheme. Clin Chem.

[13] Glendenning P (2006). Current assays overestimate 25-hydroxyvitamin D 3 and underestimate 25-hydroxyvitamin D 2 compared with HPLC: need for assay-specific decision limits and metabolite-specific assays. Ann Clin Biochem.

[14] Lensmeyer GL (2006). HPLC method for 25-hydroxyvitamin D measurement: comparison with contemporary assays. Clin Chem.

[15] Lips P (1999). An international comparison of serum 25-hydroxyvitamin D measurements. With Other Metab Bone Dis.

[16] Carter GD (2009). 25-Hydroxyvitamin D assays: the quest for accuracy. Clin Chem.

[17] Binkley N (2008). Correlation among 25-hydroxy-vitamin D assays. J Clin Endocrinol Metab.

[18] Binkley N (2004). Assay variation confounds the diagnosis of hypovitaminosis D: a call for standardization. J Clin Endocrinol Metab.

[19] Taylor AE, Keevil B, Huhtaniemi IT (2015). Mass spectrometry and immunoassay: how to measure steroid hormones today and tomorrow. Eur J Endocrinol.

[20] Ismail AA (2002). Interference in immunoassay is an underestimated problem. Ann Clin Biochem.

[21] Stanczyk FZ (2003). Limitations of direct estradiol and testosterone immunoassay kits. Steroids.

[22] Lee JH (2015). Discrepancy between vitamin D total immunoassays due to various cross-reactivities. J Bone Metab.

[23] Cashman KD (2015). Significance of serum 24,25-dihydroxyvitamin D in the assessment of vitamin D status: a double-edged sword?. Clin Chem.

[24] Carter GD (2016). 25-Hydroxyvitamin D assays: potential interference from other circulating vitamin D metabolites. J Steroid Biochem Mol Biol.

[25] Koren L (2012). Sample preparation and liquid chromatography-tandem mass spectrometry for multiple steroids in mammalian and avian circulation. Plos One.

[26] Abu Kassim NS, Shaw PN, Hewavitharana AK (2018). Simultaneous determination of 12 vitamin D compounds in human serum using online sample preparation and liquid chromatography-tandem mass spectrometry. J Chromatogr A.

[27] Muller MJ, Volmer DA (2015). Mass spectrometric profiling of vitamin D metabolites beyond 25-hydroxyvitamin D. Clin Chem.

[28] Mena-Bravo A, Priego-Capote F, de Castro MDL (2015). Study of blood collection and sample preparation for analysis of vitamin D and its metabolites by liquid chromatography–tandem mass spectrometry. Analytica Chimica Acta.

[29] Ogawa S (2014). Analysis of urinary vitamin D 3 metabolites by liquid chromatography/tandem mass spectrometry with ESI-enhancing and stable isotope-coded derivatization. Anal Bioanal Chem.

[30] Wang D (2022). A robust method for simultaneous measurement of serum 25(OH)D, 1,25(OH)2 D, and 24,25(OH)2 D by liquid chromatography-tandem mass spectrometry with efficient separation of 3-epi analogs, 23R,25(OH)2 D3, and 4beta,25(OH)2 D3. J Mass Spectrom.

[31] Alexandridou A, Volmer DA (2022). Sample preparation techniques for extraction of vitamin D metabolites from non-conventional biological sample matrices prior to LC-MS/MS analysis. Anal Bioanal Chem.

[32] Castillo-Peinado LdlS (2022). Measuring vitamin D3 metabolic status, comparison between vitamin D deficient and sufficient individuals. Separations.

[33] Xu S (2022). Simultaneous determination of vitamin D metabolites 25(OH)D3 and 1α,25(OH)2D3 in human plasma using liquid chromatography tandem mass spectrometry. J Mass Spectrom Adv the Clin Lab.

[34] Zou J (2022). Correlation between vitamin D metabolites and rheumatoid arthritis with osteoporosis by ultra-high-performance liquid chromatography–tandem mass spectrometry (UPLC–MS/MS). J Bone Mineral Metab.

[35] Lam V (2019). Chronic high fat feeding paradoxically attenuates cerebral capillary dysfunction and neurovascular inflammation in senescence-accelerated-murine-prone strain 8 mice. Nutr Neurosci.

[36] Agilent Technologies, I. *Reversed phase HPLC columns HC-C18(2) and TC-C18(2)*. 2022 [cited 2022 10/02/2022]; Available from: https://www.agilent.com/en/product/small-molecule-columns/reversed-phase-hplc-columns/hc-c18-2-tc-c18-2.

[37] Agilent Technologies, I. *Agilent column selection guide for HPLC*. 2022 [cited 2022 10/02/2022]; Available from: https://www.agilent.com/cs/library/applications/5991-4373EN.pdf.

[38] Yong YS (2017). A comparative study of pentafluorophenyl and octadecylsilane columns in high-throughput profiling of biological fluids. J Chin Chem Soc (Taipei).

[39] William Long and Jonathan Horton, A.T., Inc. *Analysis of positional isomers with agilent poroshell 120 PFP columns*. 2022 [cited 2022 1-/02/2022]; Available from: https://www.agilent.com/cs/library/applications/5991-4373EN.pdf.

